# Developing global image feature analysis models to predict cancer risk and prognosis

**DOI:** 10.1186/s42492-019-0026-5

**Published:** 2019-11-19

**Authors:** Bin Zheng, Yuchen Qiu, Faranak Aghaei, Seyedehnafiseh Mirniaharikandehei, Morteza Heidari, Gopichandh Danala

**Affiliations:** School of Electrical and Computer Engineering, University of Oklahoma, 101 David L. Boren Blvd, Suite 1001, Norman, OK 73019 USA

**Keywords:** Machine learning models of medical images, Global medial image feature analysis, Cancer risk prediction, Cancer prognosis prediction, Quantitative imaging markers

## Abstract

In order to develop precision or personalized medicine, identifying new quantitative imaging markers and building machine learning models to predict cancer risk and prognosis has been attracting broad research interest recently. Most of these research approaches use the similar concepts of the conventional computer-aided detection schemes of medical images, which include steps in detecting and segmenting suspicious regions or tumors, followed by training machine learning models based on the fusion of multiple image features computed from the segmented regions or tumors. However, due to the heterogeneity and boundary fuzziness of the suspicious regions or tumors, segmenting subtle regions is often difficult and unreliable. Additionally, ignoring global and/or background parenchymal tissue characteristics may also be a limitation of the conventional approaches. In our recent studies, we investigated the feasibility of developing new computer-aided schemes implemented with the machine learning models that are trained by global image features to predict cancer risk and prognosis. We trained and tested several models using images obtained from full-field digital mammography, magnetic resonance imaging, and computed tomography of breast, lung, and ovarian cancers. Study results showed that many of these new models yielded higher performance than other approaches used in current clinical practice. Furthermore, the computed global image features also contain complementary information from the features computed from the segmented regions or tumors in predicting cancer prognosis. Therefore, the global image features can be used alone to develop new case-based prediction models or can be added to current tumor-based models to increase their discriminatory power.

## Introduction

Medical imaging is commonly used in the clinical practice for cancer screening, early detection and diagnosis of tumors, prediction of cancer prognosis, and assessment of tumor response to treatment [1]. However, due to the lack of quantitative assessment tools, subjective reading and interpreting medical images by radiologists are often difficult and generate large intra- and inter-reader variability [2]. As a result, the efficacy of applying medical imaging in cancer screening and prognosis assessment is suboptimal and not robust. For example, although mammography is the most popular imaging technology used in breast cancer screening, its performance is unsatisfactory in terms of both cancer detection sensitivity and specificity [3]. Studies have shown that sensitivity of screening mammography is lower among younger women (i.e., ≤ 50 years old) [4], those who have dense breasts [5], undergo hormone replacement therapy [6], and carry certain breast cancer susceptibility genes [7]. For example, one study reported that mammography sensitivity decreased from 87.0% in women with almost entirely fatty breasts to 62.9% in women with extremely dense breasts or from 83.3% in women aged > 80 years to 68.6% in women aged < 50 years [8]. Thus, a high percentage of mammography-occult breast cancer is missed or overlooked by radiologists in reading screening mammograms. Moreover, mammographic screening generates high recall rates, and the majority of the biopsies are benign [9], resulting in potential long-term psychosocial consequences in women participating in breast cancer screening [10]. In predicting cancer prognosis or assessing tumor response to treatment, the guideline determined by the response evaluation criteria in solid tumors (RECIST) [11] used in current clinical practice often does not correlate well to the clinical outcome [12], which can generate overtreatment resulting in increased mortality and morbidity rates of the cancer patients due to unnecessary toxic side effects or aggressive surgeries [13].

To improve the accuracy and consistency in reading and interpreting medical images for cancer detection, diagnosis, and prognosis assessment, researchers have been actively working to develop and test computer-aided detection or diagnosis (CAD) schemes since the 1980s, which aim to serve as “the second reader” or provide radiologists new decision-making supporting tools [14]. Most CAD schemes include three steps: (1) detect suspicious regions that may depict tumors, (2) segment the targeted regions, and (3) train a machine learning model that fuses multiple image features computed from the segmented regions [15]. Despite great research enthusiasm and effort, false-positive detection rates of CAD schemes remain high [16], and whether using CAD can add values in clinical practice to help improve radiologists’ performance in reading and interpreting mammograms remains controversial [17]. The technical challenges and limitations in developing CAD schemes may include but not limited to (1) difficulty in accurate segmentation of the targeted tumors from the images due to tissue overlap, connection, and fuzzy boundary, which reduce the accuracy and reproducibility of the computed image features to build robust machine learning models [18]; (2) high false-positive cues in the detection schemes, which can mislead radiologists and reduce their performance [19]; (3) use of small or biased training datasets, which causes overfitting and reduces robustness of CAD schemes when applied to new testing cases [20]; (4) higher correlation of the detection results between CAD and radiologists, which reduces the clinical utility of CAD as “the second reader” [21]; and (5) difficulty in developing multi-image-based CAD schemes [22] to fuse and compare variation of the image features in the longitudinal images [23] or different views of images [24]. Thus, exploring new approaches in developing CAD schemes or machine learning models remains an unsolved but important research topic in the field of CAD-related medical imaging informatics.

Due to the difficulty in accurate tumor segmentation and identification of optimal handcrafted image features, great research effort has recently been made to apply deep learning models in developing CAD schemes [25]. Although developing deep learning models can avoid tumor segmentation, it requires “big data” (availability of large training datasets). Thus, besides working to investigate how to optimally apply the deep learning method to develop robust CAD schemes using the small image datasets [26–28], we also investigate a different conventional machine learning approach that uses global image features computed from the entire imaged organs (i.e., breast, lung, and abdominal region) to train prediction models without suspicious region or tumor segmentation (as used in the conventional tumor-based schemes) or predefine the regions of interests with a fixed size (as used in many deep learning-based schemes). The new global image feature analysis-based models can be either implemented to build new case-based CAD schemes or fused with the existing tumor-based CAD schemes. The hypothesis of this new approach is based on the scientific premise and preliminary study results reported in the literature, which show that image features computed from background parenchymal or specific non-tumor regions also contain high discriminatory information to help predict cancer risk [29] and prognosis [30].

To test our hypothesis, we conducted several studies to develop and test a variety of new machine learning models using or adding global image features to predict cancer risk and cancer prognosis after surgery or chemotherapy. This study reviews several CAD schemes implemented with the global image feature analysis-based machine learning models developed in our recent studies. These models were built using different types of medical images including full-field digital mammography (FFDM), magnetic resonance imaging (MRI) and computed tomography (CT) images for breast, lung, and ovarian cancers. Moreover, to demonstrate the robustness of this new concept and the developed models, new experiments and data analysis results are also included in this study. Specifically, the basic concept or structure of the models is presented in Section 2, the new experiments and data analysis results are reported in Section 3, and the unique characteristics of this new approach and future research directions are discussed in Section 4 of this paper.

## New quantitative imaging models

### Prediction of short-term breast cancer risk

Breast cancer is the most prevalent cancer in women. Detection of invasive cancer at an early stage plays an important role in cancer treatment and reduction of patients’ mortality rates. However, due to the extremely low cancer detection yield (i.e., ≤ 0.3–0.5%) and higher false-positive recall rate (i.e., ≥ 10%), the efficacy of current population-based breast cancer screening using medical imaging (i.e., mammography with or without adjunction of ultrasound or MRI [31]) is quite low. To address and overcome this issue, developing a new risk-based breast cancer screening paradigm has been attracting research interest [32]. The objective of the new approach is to develop a new model to predict short-term cancer risk, which enables to stratify the general screening population into two groups. Women assigned to the higher-risk group should be screened in a short interval (i.e., annual screening) after the negative screening of interest, while women assigned to the low-risk group can be screened in a longer interval until their short-term risk is significantly increased in the future reassessment. However, the current breast cancer risk factor or prediction models based on the epidemiology studies do not have discriminatory power to predict short-term risk in a woman developing breast cancer after a negative screening of interest and help a woman or her physician decide the optimal screening interval and/or method [33].

In our studies, we developed and tested a new risk prediction model based on the following scientific evidence or experimental observations: (1) humans naturally show bilateral symmetry in paired morphological traits, including the breasts; (2) bilateral asymmetry of breast tissue patterns is an important imaging phenotype or marker associated to biological processes; (3) bilateral mammographic tissue asymmetry and its change over time are commonly assessed by the radiologists in their decision-making process of cancer detection; and (4) CAD schemes can yield more consistent results in quantifying mammographic density and their bilateral asymmetry by avoiding inter-reader variability [34]. Thus, we build a new model to predict short-term breast cancer risk based on the computation and analysis of bilateral mammographic density and tissue asymmetry of the negative images between the left and right breast. The goal is to predict the likelihood of a woman developing detectable cancer in the short term (i.e., the next subsequent annual mammographic screening).

**Fig. 1:**
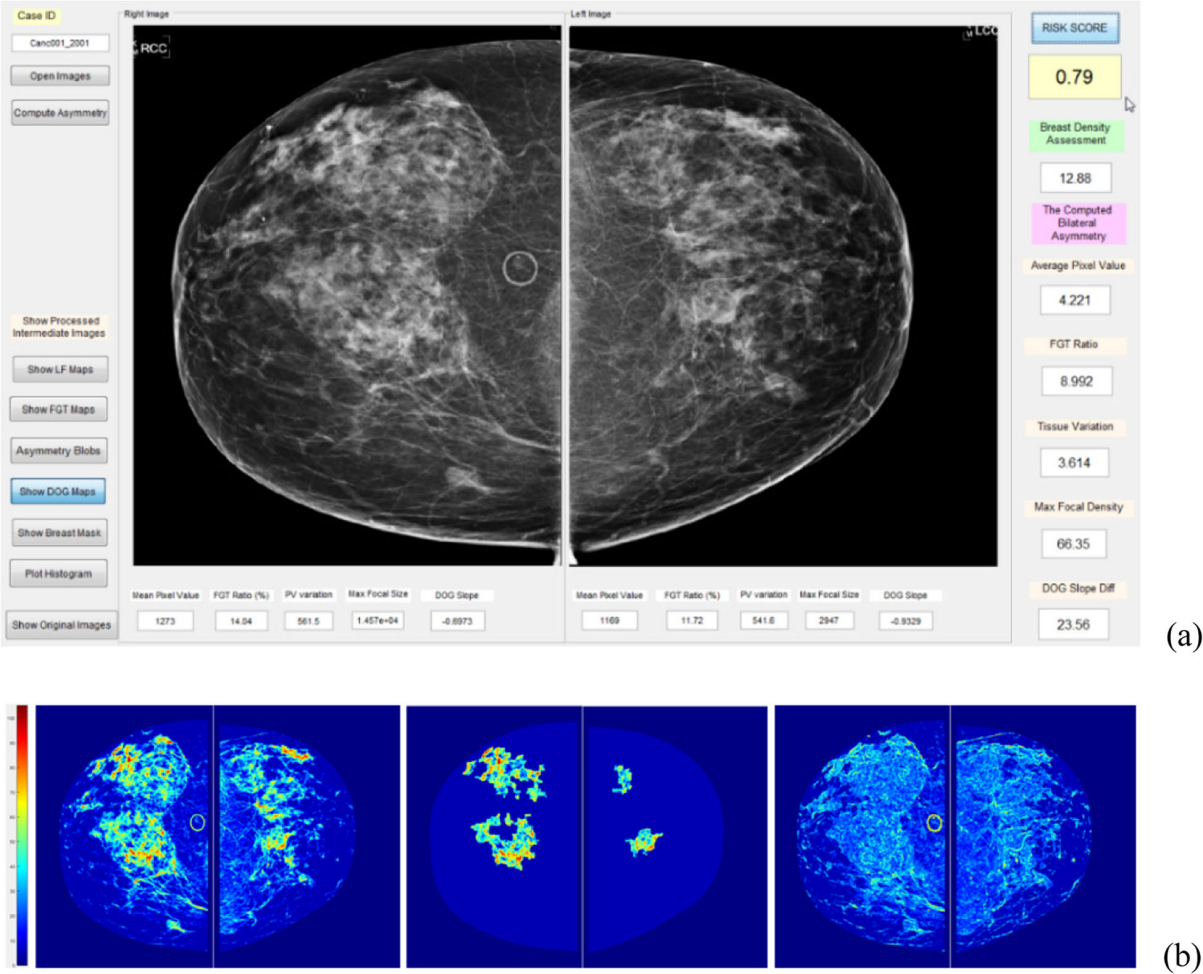
The GUI of a CAD-based short-term breast cancer risk model (**a**) and bilateral asymmetry of dense tissue regions, local focal regions, and local pixel value fluctuation maps from the left to right (**b**)

Figure 1 shows a graphic user interface (GUI) of the prediction model. Once two bilateral negative mammograms of either cranio caudal (CC) or mediolateral oblique (MLO) view are loaded into the GUI, a user can click the button on the top-left corner of the GUI to order the scheme processing the images and computing features (${F}_i^L$ and ${F}_i^R$) between the left and right mammograms and their difference ($\Delta  {F}_i=\left|{F}_i^L-{F}_i^R\right|,i=1,\cdots, n$). Once the required features are computed and displayed, the user can view several asymmetrical patterns or maps (Fig. 1b) and click the “Risk Score” button on the top-right corner of the GUI (Fig. 1a). A short-term cancer risk score (ranging from 0 to 1) of the case is displayed. In the example case (Fig. 1), the model-generated risk score is 0.79. The higher score indicates a higher risk of having or developing breast cancer in the short term. The details of developing this model to predict short-term breast cancer risk have been reported in our previous study [34].

### Prediction of lung cancer recurrence risk

Recently, promoting and implementing lung cancer screening programs using low-dose CT imaging and other incident findings in detecting or diagnosing chromatic lung diseases have resulted in detecting more early-stage lung cancers. Most of the cases are non-small-cell lung cancers (NSCLCs). Although early cancer detection and surgical treatment help reduce the mortality rate of patients diagnosed with early-stage NSCLC, lung cancer recurrence rates after surgery of tumor resection is still high (i.e., ranging from 30% to 60% as reported in the literature [35]). To more accurately stratify patients into two groups of having a high and low risk of cancer recurrence, researchers have investigated many genomic biomarkers to predict the risk of cancer recurrence in patients with early-stage NSCLC [36]. Patients with high cancer recurrence risk need to be continuously treated after surgery using other methods (i.e., radiation therapy or chemotherapy) to reduce the risk of cancer recurrence and increase the cancer-free survival.

**Fig. 2:**
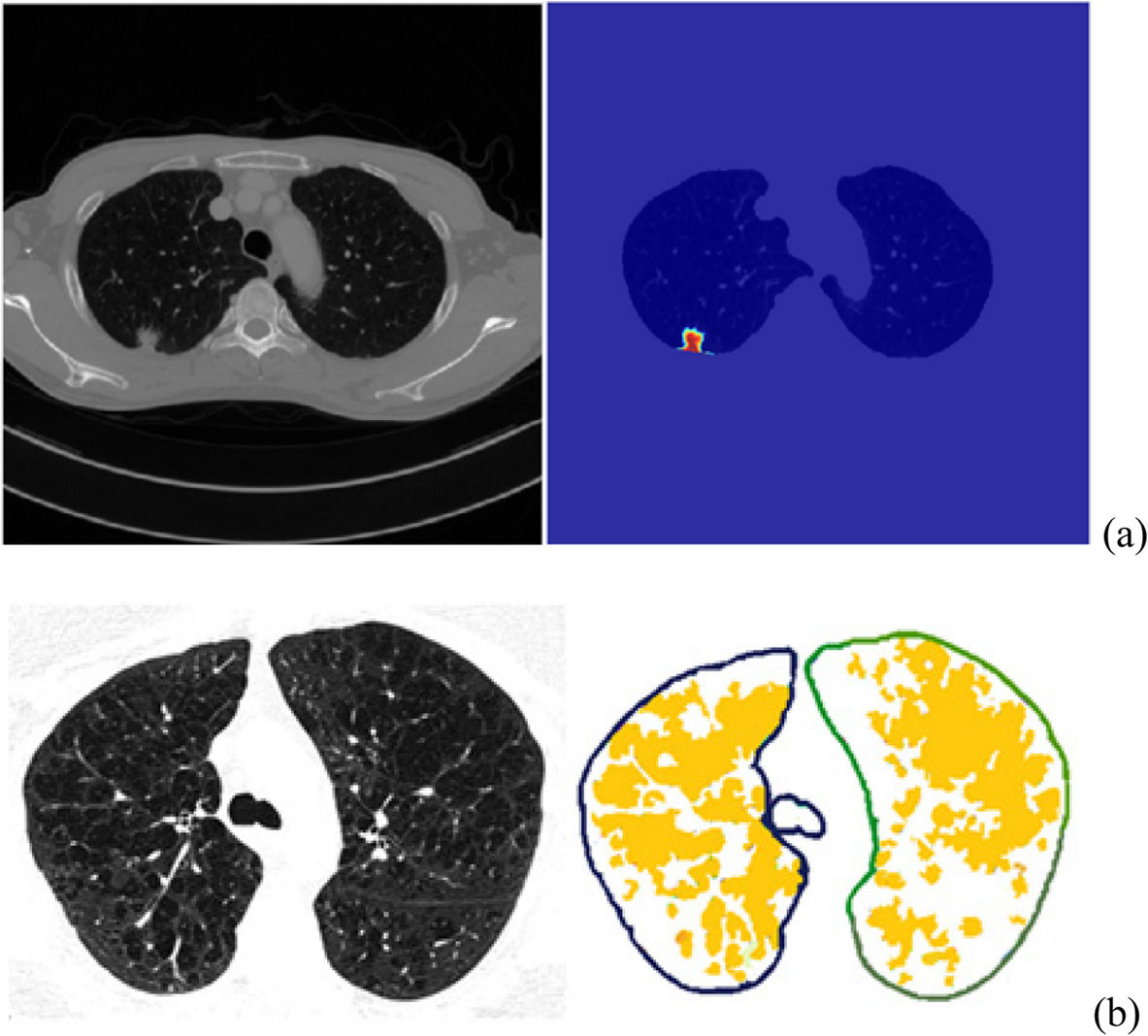
Illustration of segmenting lung tumor marked by red color and bright boundary (**a**) and segmenting emphysema regions marked by yellow color using a density mask (**b**)

In addition to the genomic biomarkers, CT images also contain useful information in predicting the prognosis of patients with NSCLC. For example, chronic obstructive pulmonary disease (COPD) is another well-recognized higher risk factor of developing lung cancer, and emphysema is one of the most important symptoms of COPD. In our study, we investigated whether the global emphysema-related image features include useful information or discriminatory power to predict the risk of lung cancer recurrence. We developed and tested a new CAD scheme implemented with a machine learning model to combine tumor-related image features computed from the segmented lung tumors and global emphysema-related image features computed from the entire lung volume depicted on CT images. In tumor segmentation, the scheme applied a modified region growing algorithm controlled by a convex hull function to stop the leakage of the segmented tumor region to the normal lung tissues and smooth the segmented tumor boundary [37]. The CAD model using tumor-based image features have been trained and tested in our previous studies [37, 38]. In the recent study, the new scheme applies a density mask (using the threshold of ≤ − 950 HU) to automatically segment and quantify the percentage of emphysema blobs or regions. Figure 2 presents an example of the segmentation of lung tumor and emphysema blobs in one CT image slide. From the image processing results, a set of both tumor-based and global emphysema-based image features are computed and then used to train machine learning models to predict lung cancer recurrence risk. The details of testing this new model will be described in subsection 3.2 of this paper.

### Prediction of breast cancer response to neoadjuvant chemotherapy

Currently, neoadjuvant chemotherapy has been increasingly used as first-line therapy in patients diagnosed with locally advanced breast cancer. Previous studies have demonstrated long-term prognosis among the high proportion of patients who have good post-treatment imaging responses, such as those who have a pathologic complete response (pCR) at the time of surgery. However, due to the lack of accurate prognostic markers, a higher percentage of patients with pCR still undergo unnecessary and aggressive surgery. Additionally, other patients who do not respond to chemotherapy suffer from unnecessary toxic side effects. Such overtreatment increases patients’ morbidity and mortality rates [39]. As a result, many research groups have attempted to develop CAD schemes of breast MRI in assessing tumor response to chemotherapies by comparing the changes of the contrast-enhanced kinetic image features computed from the tumors segmented from the MR images obtained before and after chemotherapy.

**Fig. 3:**
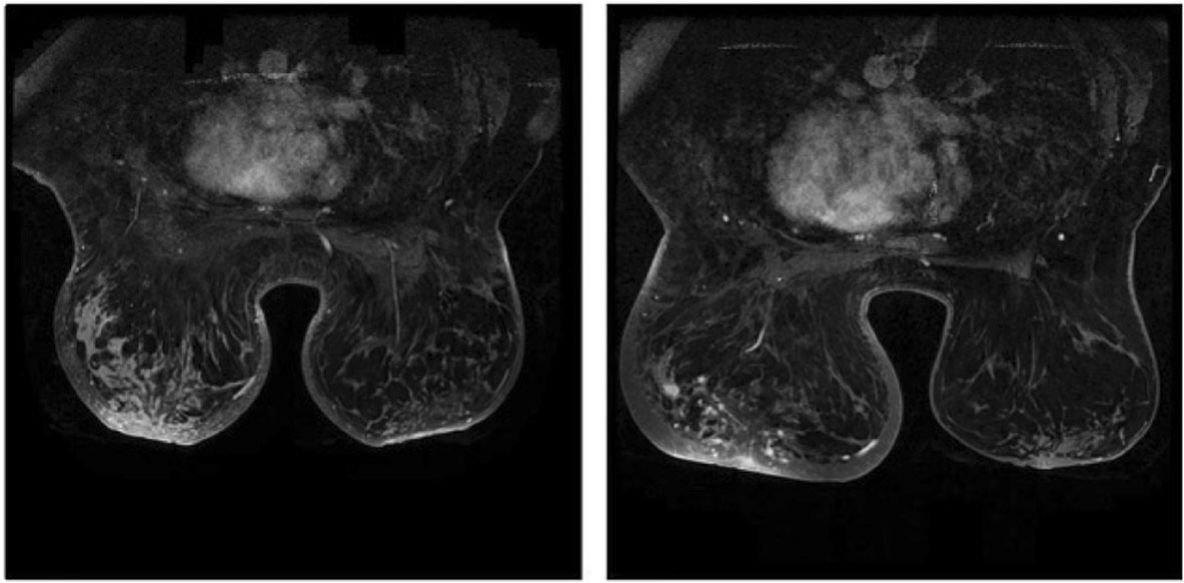
Diffused tumors enhanced in breast MRI performed before (left image) and after (right image) neoadjuvant chemotherapy

**Fig. 4:**
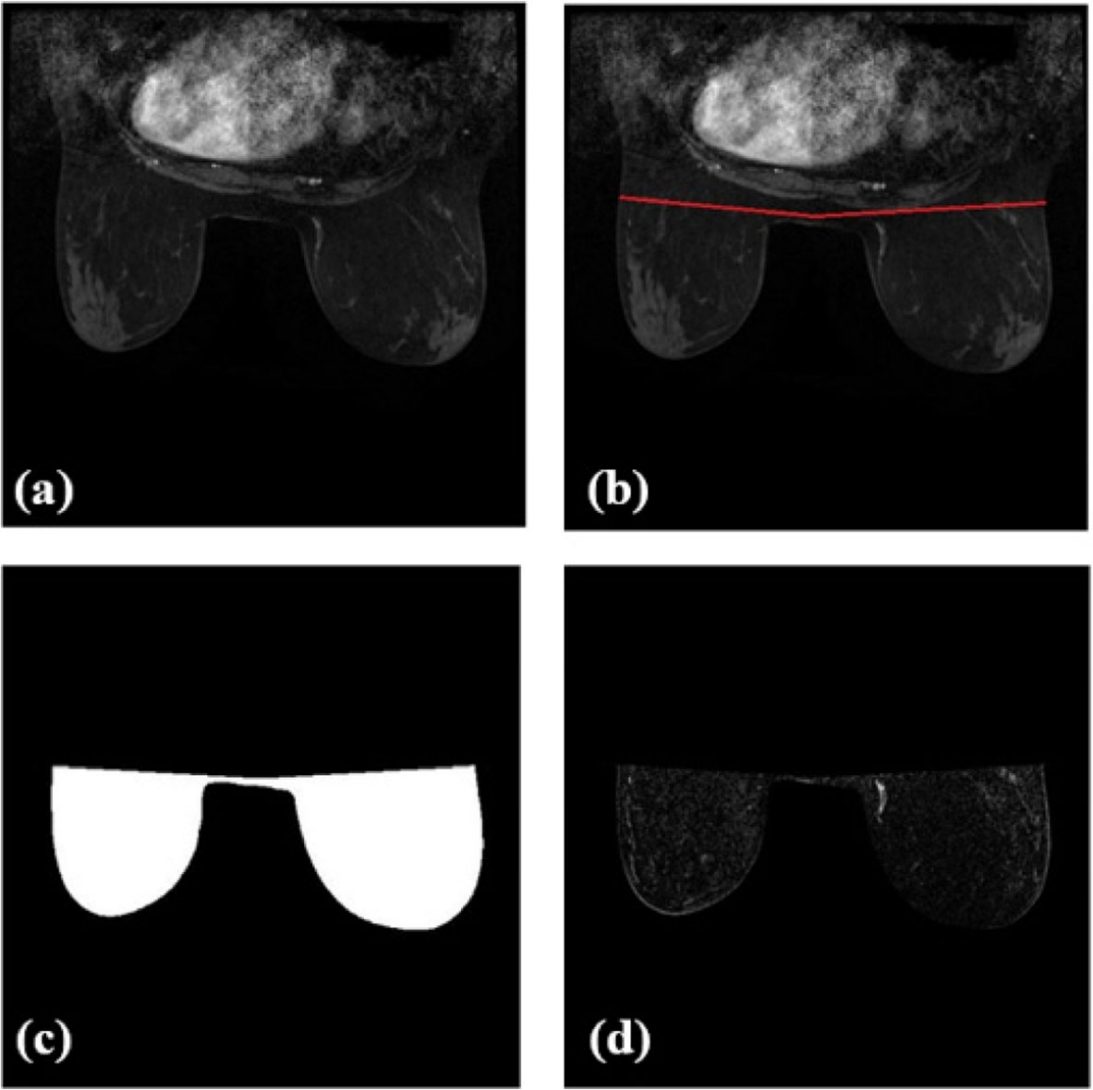
Breast region segmentation steps and generating the contrast-enhanced image map including (**a**) the original image, (**b**) separation line, (**c**) generated mask, and (**d**) breast region  segmented on the contrast-enhanced map

In our study, we found that many cases include tumors with diffused enhancement (Fig. 3). Accurately defining and segmenting the diffused tumors are difficult and often unreliable. Thus, we built a new model based on the analysis of globally kinetic breast MRI features to predict tumor response (i.e., complete response based on the RECIST guidelines) to neoadjuvant chemotherapy using breast MRI acquired before chemotherapy only. From the MR image, a CAD scheme is applied to segment breast area by removing all pixels in the air background and behind pectoralis muscle (Fig. 4). Then, the CAD scheme performs image registration and subtraction of two sets of the matched breast MRI slices acquired in two MRI sequence scans performed before and after injection of gadopentetate dimeglumine contrast agent. After generating the contrast enhancement maps of the breast area, the CAD scheme computes a set of global kinetic image features. Specifically, the features include the mean, standard deviation, and skewness of the contrast enhancement values computed from all pixels inside the segmented breast volume, which represent the magnitude and heterogeneity of contrast enhancement of the global area. Next, the CAD scheme sorts the contrast enhancement values from the maximum to the minimum and computes two new features representing the average contrast enhancement value among the pixels listed in the top 1% and top 5% of the sorting list. Lastly, to overcome the impact of heterogeneity of background parenchymal enhancement in different patients, the scheme also computes asymmetrical image features that represent the bilateral differences of two kinetic image feature values, which are computed from MRI of the left and right breasts. As a result, without tumor segmentation, this new model trained using global image features is applied to process all breast MRI depicting either solid or diffused tumors and predict their response to neoadjuvant chemotherapies. The details of developing and testing this model have been presented in our previous study [40].

### Prediction of chemotherapy efficacy in patients with ovarian cancer

Ovarian cancer has the highest mortality rate in gynecologic malignancy. Most ovarian cancers detected in the clinical practice (> 85%) are epithelial ovarian cancers (EOCs) and they are typically diagnosed in the advanced stage with metastatic tumors spreading to other organs of the body. In these patients, angiogenesis plays a fundamental role in the pathogenesis of EOC, which results in higher vascular endothelial growth factor expression and promotes tumor growth, ascites, and metastases. Thus, new chemotherapies (i.e., bevacizumab) that target the angiogenesis-specific pathways were developed and tested in many clinical trials. However, studies have shown that some patients received benefits with the increased progression-free survival (PFS) or overall survival (OS), while others did not receive benefits due to the high toxicity and other serious side-effects [41]. How to effectively identify patients with EOC who are most likely to benefit from receiving bevacizumab or other antiangiogenic therapies remains an unsolved clinical issue in the treatment of patients with EOC. Thus, identifying effective imaging markers and/or developing prediction models can help address or solve this clinical issue.

**Fig. 5:**
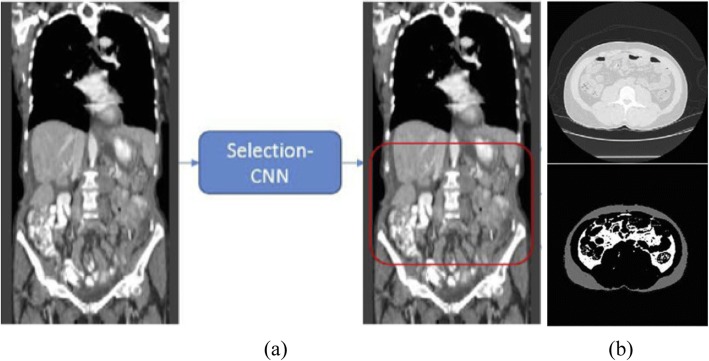
Applying a convolutional neural network (CNN) algorithm (**a**) to automatically identify the targeted abdominal region marked inside a red frame and segmenting each selected abdominal CT slice into three groups of pixels, namely, SFA (light gray), VFA (white), and other human organs (dark) (**b**)

**Fig. 6:**
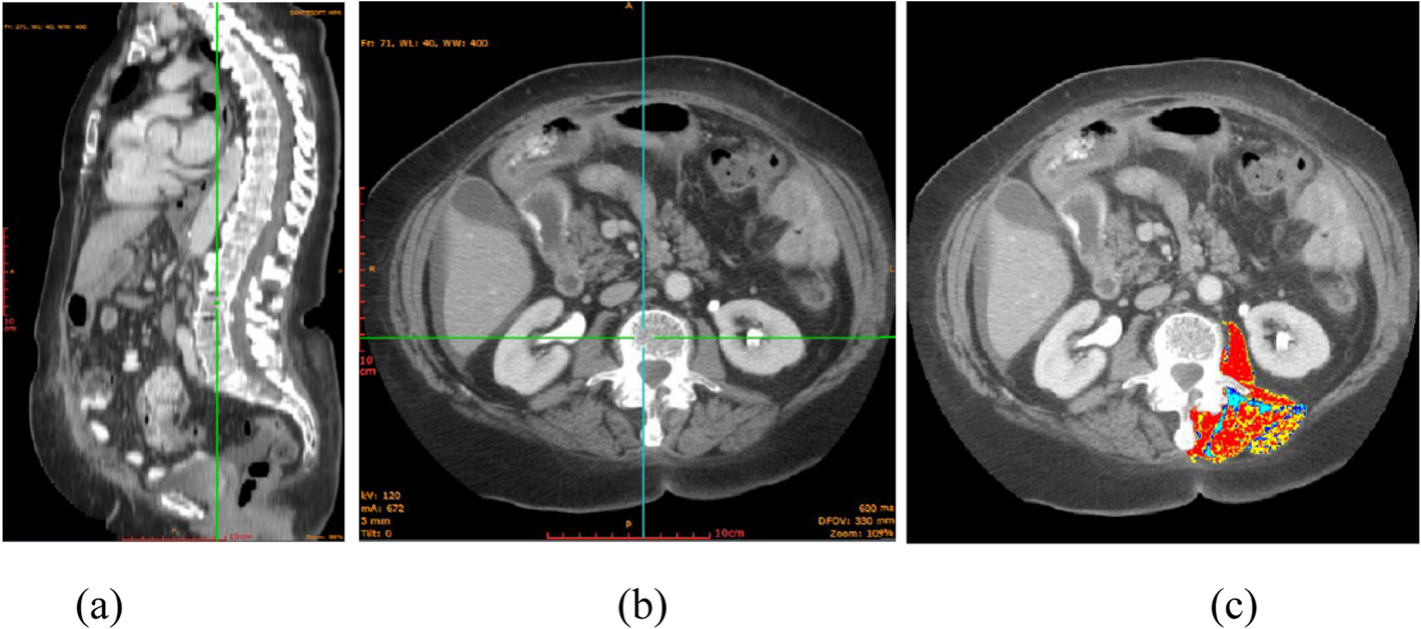
Identifying the level (L3) of vertebral spines in (**a**) the sagittal and (**b**) axial views and (**c**) the segmented TPA and its surrounding muscle region where the color indicates the heterogeneity of muscle density

In addition to developing CAD schemes with machine learning models trained using image features computed from the targeted tumors based on the RECIST guidelines [42], we also investigated and built CAD models and GUI to process abdominal CT images acquired from patients with EOC before performing chemotherapy, segment the targeted non-tumor regions, compute image features, and train the machine learning model to predict PFS or OS in patients receiving bevacizumab-based chemotherapy. The first set of image features is computed to quantify the adiposity of patients [43]. As shown in Fig. 5, a convolution neural network is applied to identify CT image slices within the abdominal region. Then, the CAD scheme is applied to process all selected CT slices and segment pixels inside the abdominal region into three groups using a fat threshold range of − 140 to 40 HU, which represents visceral fat area (VFA), subcutaneous fat area (SFA), and other human organs depicted on CT images. From the segmented VFA and SFA, the scheme computes image features to quantify the volume and heterogeneity of the adiposity characteristics. The second set of image features is computed to quantify the size and density heterogeneity of the total psoas area (TPA) and its surrounding muscle region (as shown in Fig. 6) [44]. Using these global and targeted non-tumor-related image features, we trained machine learning models to predict the outcome of PFS and OS in the patients with EOC. The details of the model development have been reported in our previous study [30].

### Steps in training machine learning models using small datasets

Although we can compute a large pool of image features (i.e., morphological, density heterogeneity, and texture) from the original medical images or transformed maps (i.e., frequency domain), identifying small sets of optimal image features from the initially large feature pools is an important and challenging task to improve the performance and robustness of the multiple image feature fusion-based machine learning models. Additionally, we also often encounter two difficult issues related to image datasets. The first is the relatively small number of cases, and the second is the unbalance between two case classes (i.e., more negative cases than positive cases). Thus, to minimize or reduce case selection bias in searching for optimal features and training machine learning models in our studies, we perform the following model training and testing steps.

First, a synthetic minority oversampling technique (SMOTE) [45] is applied to balance the number of cases in two classes to achieve the ratio close to 1:1 (if needed). The details of applying the SMOTE algorithm in our model development have been reported in several studies (i.e., ref. [38]). Second, a feature selection algorithm (i.e., modified sequential forward floating selection [46]) or feature regeneration algorithm (i.e., locality preserving projection [47]) is applied to search for and build an optimal feature set or vector. Third, a machine learning model (i.e., artificial neural network and support vector machine) is trained and tested. Because of the availability of the relatively small image dataset, we typically apply a cross-validation method, such as a 10-fold or leave-one-case-out (LOCO) cross-validation method, to train and test the model. When using a cross-validation method, the first and second steps of SMOTE and feature selection are embedded into the cross-validation loop to train and test the model. Thus, the testing cases will be excluded from the above two processes of generating synthetic data and feature selection or regeneration. Each testing case (including the synthetic case) is tested once and receive one prediction score (ranging from 0 to 1) generated by the trained machine learning model. Lastly, the synthetic data or cases are removed, and then the area under a ROC curve (AUC) or adjusted odds ratio (OR) is used as evaluation indices to assess model performance. Furthermore, by applying an operation threshold on the model-generated risk scores, we build a confusion matrix to compute the overall prediction accuracy (i.e., sensitivity, specificity, and positive predictive value).

## Experiments and results

During the last several years, we have conducted several experiments to test our new machine learning models based on the analysis of global or non-tumor-related image features. The experimental and data analysis results have been reported in several previous papers, such as prediction of the following:

1. Short-term breast cancer risk using the bilateral mammographic density asymmetrical features computed from the “prior” negative screening mammograms [34, 47, 48];
2. Likelihood of the case being abnormal using the global image features computed from the “current” screening mammograms (case-based CAD scheme) [16, 49];
3. Response of breast tumors to neoadjuvant chemotherapies using the global kinetic image features computed from the breast MRI performed before chemotherapy [40];
4. Response of ovarian cancer patients to chemotherapy using the global adiposity-related image features computed from abdominal CT images performed before chemotherapy [30, 42].

In this study, we report two new sets of experiments and data analysis results, which have not been previously published in the peer-reviewed journal papers.

### Prediction of short-term breast cancer risk

A retrospectively assembled image dataset, which involves images acquired from 1045 women who underwent at least two annual mammographic screening, was used in one recent study. Specifically, each case had two subsequent screenings defined as “current” and “prior” screenings with a time interval ranging from 12 to 18 months. All “prior” images were detected as negative by the radiologists in the original mammography screening. In “current” screenings, cancers were detected and confirmed in 402 cases, while the remaining 643 remain negative. Each screening mammography has 4 images in the CC and MLO view of the left and right breasts. Thus, all “prior” negative mammograms were selected and processed by the model to predict the risk or likelihood of developing cancer that is detectable in the next (“current”) mammographic screening.

The CAD scheme computes six image feature differences between the left and right mammograms. Briefly, from one bilateral pair of either CC or MLO view images and the processed or transformed image maps, the scheme computes bilateral difference of (1) the average mammographic density, (2) fibro-glandular tissue volume, (3) size of the CAD-detected focal asymmetric regions, (4) average pixel values computed from the two local breast tissue fluctuation maps, (5) average pixel values computed from the maps generated using a difference-of-Gaussian filter, and (6) overall mammographic density (similar to the breast imaging reporting and data system [BIRADS]).

The CAD scheme then uses a k-nearest neighborhood (KNN) model, which fuses above six image features to generate a risk score to predict the likelihood of cancer being detected in the next subsequent mammographic screening. First, the similarity is assessed by the difference in feature values, *f*_*r*_(*x*), between a queried case (*y*_*q*_) and reference case (*x*_*i*_) in a multi-dimensional (*n*) feature space,

$$ d\left({y}_q,{x}_i\right)=\sqrt{\sum \limits_{r=1}^n{\left[{f}_r\left({y}_q\right)-{f}_r\left({x}_i\right)\right]}^2} $$

Next, a distance weight (*w*_*i*_) is defined as

$$ {w}_i=\frac{1}{d{\left({x}_q,{x}_r\right)}^2} $$

Lastly, the cancer risk prediction score is computed as

$$ {P}_{risk}=\frac{\sum_{i=1}^N{w}_i^{Pos}}{\sum_{i=1}^N{w}_i^{Pos}+{\sum}_{j=1}^M{w}_j^{Neg}} $$

in which the total reference cases compared are *K* = *N* + *M*. Two weighting factors, ${w}_i^{Pos}$ and ${w}_j^{Neg}$, are the computed distance of the positive and negative cases in the “current” mammographic screening. In this KNN-based prediction model, K = 15.

Using a LOCO cross-validation method, this risk model yielded a prediction performance of AUC = 0.70 ± 0.02. Table 1 presented the relative adjusted odds ratios (ORs) and corresponding 95% CI. It shows that, by dividing the model-generated prediction scores into five subgroups with an approximately equal number of cases, five adjusted ORs monotonically increase from 1.0 to 8.13. The logistic regression analysis also indicates an increasing trend with statistical significance as the increase of model-generated prediction scores (*p <* 0.01). For a comparison, when dividing the cases into four BIRADS bins, four adjusted ORs are computed in a range of 1.0 to 1.27. The corresponding logistic regression analysis does not show an increasing or decreasing trend (*p =* 0.346). Thus, the results support that although mammographic density rated using BIRADS is a well-known breast cancer risk factor, it cannot be used to predict short-term breast cancer risk [33]. Our new model-generated scores are different from BIRADS ratings of mammographic density, which yielded significantly higher discriminatory power in predicting short-term breast cancer risk.

**Table 1:** Comparison of the adjusted odds ratios (OR) and 95% CIs between the new model-generated risk scores and mammographic density ratings by the radiologists

Risk factor	Subgroup	Number of cases	Adjusted ORs	95% CI
New prediction model	1	34–175	1.00	Baseline
	2	62–147	2.17	[1.35, 3.48]
	3	84–125	3.46	[2.18, 5.48]
	4	94–115	4.21	[2.66, 6.65]
	5	128–81	8.13	[5.13, 12.9]
Density BIRADS	1	21–40	1.00	Baseline
	2	158–250	1.20	[0.69, 2.12]
	3	218–328	1.27	[0.73, 2.21]
	4	5–25	0.38	[0.13, 1.14]

### Prediction of lung cancer recurrence risk

A retrospective dataset involving 107 patients diagnosed with early-stage NSCLC was used in this experiment. Postoperatively, 26 patients had a cancer recurrence, while 81 had disease-free survival (DFS) in 3 years. A CAD scheme was applied in processing CT images of these patients, which were acquired preoperatively, and compute to a pool of 56 image features, which includes 35 tumor-related morphological, CT number distribution, and texture features computed from the segmented three-dimensional tumor volume as reported in our previous study that built a prediction model using tumor-related image features only [37] and 21 emphysema-related features computed from the entire lung volume of the CT images. These 21 features are divided into three subgroups representing emphysema volume and shape, density distribution and heterogeneity, and gray-level texture-based features.

First, due to unbalanced data (26 positive cases for cancer recurrence and 81 negative cases for DFS in 3 years), a SMOTE algorithm was applied to add synthetic data and double the “positive” test cases from 26 to 52 to improve case balance in the two classes. Thus, a total of 133 cases were used to build and optimize random forest tree models. Second, a correlation-based feature selection (CFS) algorithm implemented in Weka data mining software package with a best-first heuristic feature selection criterion [50] was applied to select a subset of optimal features from the initial feature pool. This feature selection method evaluates the value of a subset of features with respect to the discriminative power of each individual feature along with the degree of redundancy between the features. Using the LOCO cross-validation method, we sorted the frequency of the selected top performed features and finally assembled a small and optimal set of eight image features, which include five tumor-related features and three emphysema-related features. Lastly, we built and compared three random forest models using five tumor-related features, three emphysema-related features, and all eight features. The LOCO cross-validation method was used to train the model and validate its performance.

The selected three top-performing emphysema-related features are entropy, autocorrelation, and uniformity of global emphysema patterns. AUC values using these three individual features to predict the risk of lung cancer recurrence are 0.63 ± 0.07, 0.57 ± 0.06 and 0.66 ± 0.06, respectively. It indicates that, unlike the subjective reading of radiologists, which estimates a percentage of emphysema regions over the entire lung volume, the CAD scheme can detect and quantify more image features that have higher or improved discriminatory power than the percentage of emphysema in the total lung volume. By comparing these three random forest tree models trained using tumor-related features, emphysema-related features, and combined features, AUC values are 0.79 ± 0.05, 0.70 ± 0.07, and 0.84 ± 0.03, respectively, which indicates that adding global emphysema-related features significantly increases prediction performance (*p <* 0.05).

After applying an operation threshold of T = 0.5 to the model-generated prediction scores, we assembled three confusion matrices (Table 2). From the confusion matrices, we computed and compared the prediction performance of three models (Table 3). The results indicate that we can identify global emphysema-related image features that do not only have reasonable discriminatory power but also are complementary to tumor-related image features. Thus, adding global image features into the machine learning model improves the performance of predicting the risk of lung cancer recurrence in patients with early-stage NSCLC postoperatively. Specifically, by fusing image features computed from the segmented tumor and global emphysema regions, Model 3 increases the overall accuracy of predicting cancer recurrence risk by more than 10% to 83.2% as comparing to Models 1 and 2.

**Table 2:** Comparison of three confusion matrices generated by three machine learning models trained using tumor-related features (Model 1), emphysema-related features (Model 2), and combined image features (Model 3) to predict the risk of lung cancer recurrence

Cancer recurrence	Model 1		Model 2		Model 3	
	Yes	No	Yes	No	Yes	No
Prediction – yes	13	14	15	19	18	10
Prediction – no	13	67	11	62	8	71

**Table 3:** Comparison of prediction performance of three machine learning models trained using tumor-related features (Model 1), emphysema-related features (Model 2), and combined image features (Model 3)

Performance index	Model 1	Model 2	Model 3
Sensitivity	50.0%	57.7%	69.2%
Specificity	82.7%	76.5%	87.7%
Positive predictive value	48.1%	44.1%	64.3%
Negative predictive value	83.8%	84.9%	89.9%
Overall accuracy	74.8%	72.0%	83.2%

## Discussion

To help establish new precision or personalized medicine, identifying new genomic biomarkers and quantitative imaging markers and developing the multiple feature fusion-based machine learning models have been attracting broad research interest in the biomedical research field recently. Based on the newly proposed radiomics concept and several preliminary studies, it is feasible to identify and compute quantitative medical imaging markers to assist the prediction of cancer risk and prognosis. Since medical imaging is commonly used in clinical practice, developing new prediction models based on medical image features provides an unprecedented opportunity to support radiologists, oncologists and/or surgeons in their decision making of cancer diagnosis and treatment at low cost [51]. Recently, we have been exploring and developing several new CAD-supported machine learning models. These models can be applied to radiographic images of human bodies (i.e., presented in this paper) or animal models [52] and to digital histopathology images [28]. In this study, we reviewed several prediction models trained using global or non-tumor-related image features computed from a variety of medical images (FFDM, MRI, and CT) for breast, lung, and ovarian cancers with new experimental results. From our recent studies, we observed several unique characteristics of developing new image processing and machine learning models involving global or non-tumor-related image features.

First, although many cancer risk factors have been identified and used in existing cancer risk prediction models based on epidemiology studies, these models lack the clinically accepted discriminatory power to help establish new risk-based or personalized cancer screening programs [33]. For example, breast MRI has the highest cancer detection sensitivity [31] and has been recommended by the American Cancer Society as an adjunct screening tool to mammography in women with increased cancer risk (i.e., > 20–25% of lifetime risk). However, it excludes most women who have mammography-occult early breast cancers. In contrast, annual MRI screening in small groups of women at the elevated risk has a quite low cancer detection yield (i.e., 2–3%) [53]. However, quantitative imaging markers are time-dependent, which makes them significantly different from most genetic and lifestyle-based risk factors used in existing cancer risk models. Thus, the risk scores generated by imaging markers or machine learning models will increase as the time interval to having or developing image detectable cancer shortens [34]. As a result, cancer risk prediction models based on quantitative image features have advantages in predicting short-term cancer risk after having negative screening of interest, which can help stratify the general cancer screening population into different groups with variable screening intervals to improve efficacy of cancer screening (i.e., increase cancer detection yield and reduce unnecessary biopsies of benign cases). Since medical image features vary as cancer risk increases or decreases, it has the potential to establish different screening intervals or strategies for individuals at different life periods. In our studies, we observed that, although tumors are not detectable in the negative screening images, the global image features computed from these images carry useful information or markers to predict cancer risk. For example, we demonstrated the advantages of developing short-term breast cancer risk prediction models based on bilateral mammographic density and tissue pattern asymmetry. A similar concept may also be applied in screening other types of cancer. For example, based on the current guideline issued by the US Preventive Services Task Force, annual screening for lung cancer using low-dose CT images only applies to adults aged 55–80 years who have a 30 pack-year smoking history and currently smoke or have quit smoke within the past 15 years. This guideline also has similar weaknesses of low cancer detection yield among the targeted smoking groups and omitting the majority of nonsmokers who can also develop lung cancer. To address this challenge, researchers can also develop new short-term lung cancer risk prediction models using the quantitative image features computed from the negative lung CT images, such as using the features related to the heterogeneity of COPD patterns [54].

Second, the most current CAD schemes are tumor-based schemes aiming to detect suspicious tumors, classify malignant and benign tumors, and predict or assess tumor response to chemotherapies. The challenges of using these tumor-based CAD schemes include (1) high false-positive rates, which may impose a negative impact on radiologists and reduce their image reading performance [17, 19], and (2) difficulty and error in tumor segmentation, which reduces the accuracy and robustness of the computed image features [18]. The case-based CAD schemes only use global image features without detecting tumor locations and segmenting tumor regions. This makes developing global image feature analysis model-based CAD schemes simpler and probably more robust. However, the new case-based CAD schemes do not directly compete with tumor-based CAD schemes. For example, although case-based CAD schemes may not be used as “a second reader” as the current tumor-based CAD schemes, they have the potential to be used as prescreening tools to help stratify image cases into high- and low-risk groups (e.g., like prescreening performed by technologists [55]). Using the model-generated prediction scores (or “warning” signs), radiologists can focus on reading and interpreting higher risk cases to increase detection sensitivity by reducing the risk of missing or overlooking subtle tumors, while reducing image reading time in lower risk cases. Thus, adding this prescreening process may help improve both the accuracy and efficiency of radiologists in reading and interpreting medical images in the busy or high-volume clinical practice.

Third, our studies also demonstrated that the models developed using global image features can not only generate higher or equivalent discriminatory power compared to the conventional tumor-based models but also provide complementary information due to the lower correlation between the image features and prediction scores generated by these two types of models. Thus, an optimal fusion of quantitative image features computed from both the tumor and global parenchymal regions can further improve model performance in detecting suspicious breast tumors [49] and predicting the risk of lung cancer recurrence (Tables 2 and 3). Such a fusion approach can also be expanded to optimally combine imaging markers and genomic biomarkers to improve model performance in cancer risk prediction, tumor diagnosis, and prognosis assessment [37, 56].

Fourth, the efficacy of cancer treatment (i.e., using chemotherapies) depends on not only the characteristics of tumors but also overall health issues of patients. Thus, it is also important to identify new imaging features or markers computed from other non-tumor regions. For example, we demonstrated that using a logistic regression model built by quantitative image features of adiposity can yield significantly higher accuracy than using the bone mass index to predict the benefit in patients with ovarian cancer who received bevacizumab-based chemotherapy [30]. Our machine learning model combining the computed image features associated with SFA, VFA, and TPA has been applied to analyze image data acquired from a large US national clinical trial (GOG 218), which involves > 1800 patients diagnosed with ovarian cancer. The study result supports the feasibility of using these non-tumor-related imaging markers as new prognostic prediction markers [57].

Therefore, this study reviewed several machine learning models based on the analysis of image features computed from global or non-tumor regions and presented new experimental data. This is a new research direction in CAD-related medical imaging informatics field, which opens an opportunity for researchers to explore new research and application tasks. For example, we recently investigated the feasibility of developing a new CAD scheme based on global mammographic image features to classify malignant and benign cases in which suspicious tumors have been detected by radiologists. The scheme initially computes 59 global mammographic image features, followed by applying a particle swarm optimization algorithm to search for optimal features and training a support vector machine model to predict the likelihood of malignancy. When using a relatively small dataset involving 134 malignant and 141 benign cases, the model yields a performance of AUC = 0.79 ± 0.07 [58], which is highly comparable to the performance of applying tumor-based CAD schemes in classifying malignant and benign tumors [26].

**Fig. 7:**
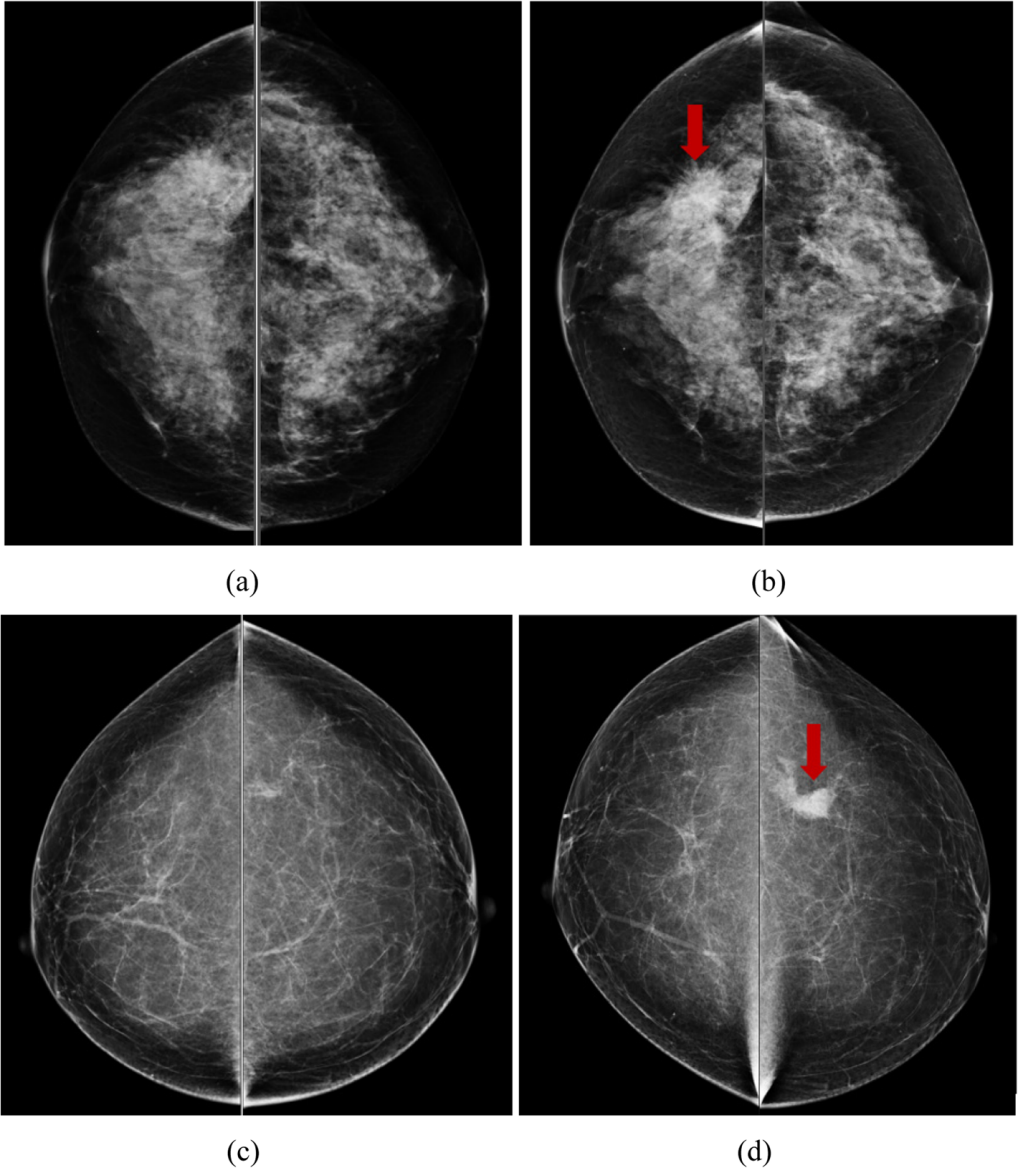
Prediction results of applying our short-term breast cancer risk models to “prior” negative images of two cases (**a** and **c**). “Current” images of two cases in which malignant tumors were detected (red arrows) (**b** and **d**). Our model correctly predicted case (**a**) as a high-risk case but the misclassified case (**c**) as a low-risk case

Despite the encouraging results, we also recognize the limitations of these studies. First, due to the use of the relatively small image datasets, the robustness of these models has not been well tested. Second, image features explored and used in our models may not be optimal, which limits model performance. Figure 7 shows an example of applying our short-term breast cancer risk prediction model to analyze “prior” negative images of two cases. Both cases were positive in the “current” screening. The model correctly predicted one case as a high-risk case (with a prediction score of 0.83) but incorrectly predicted another case as low-risk case (with a prediction score of 0.25), which is a quite aggressive case with a large tumor developed in 1 year. Therefore, developing optimal imaging feature fusion-based machine learning models to predict cancer risk and prognosis still faces many challenges particularly in detecting aggressive cases. The more innovative research effort is needed to identify more effective image features, optimize machine learning schemes, and validate model performance and robustness using larger and independent image datasets in future studies.

## Data Availability

Not applicable.

## Abbreviations

AUC: Area under a ROC curve
BIRADS: Breast imaging reporting and data system
CAD: Computer-aided detection or diagnosis
CC: Cranio-caudal
CFS: Correlation-based feature selection
CI: Confidence interval
COPD: Chronic obstructive pulmonary disease
CT: Computed tomography
DFS: Disease-free survival
EOC: Epithelial ovarian cancer
FFDM: Full field digital mammography
GUI: Graphical user interface
LOCO: Leave-one-case-out
MLO: Mediolateral oblique
MRI: Magnetic resonance imaging
NSCLC: Non-small-cell lung cancer
OR: Odds ratio
OS: Overall survival
pCR: Pathologic complete response
PFS: Progression-free survival
RECIST: Response evaluation criteria in solid tumors
ROC: Receiver operating characteristic
SFA: Subcutaneous fat area
SMOTE: Synthetic minority oversampling technique
TPA: Total psoas area
VFA: Visceral fat area
